# Impact of COVID-19 on the access to hearing health care services for children with cochlear implants: a survey of parents

**DOI:** 10.12688/f1000research.24915.1

**Published:** 2020-07-09

**Authors:** Mohammed Ayas, Ahmad Mohd Haider Ali Al Amadi, Duaa Khaled, Ahmad Munzer Alwaa

**Affiliations:** 1 University Hospital Sharjah, Sharjah, 72772, United Arab Emirates; 2 College of Medicine, University of Sharjah, Sharjah, 72772, United Arab Emirates

**Keywords:** Pediatric hearing loss, Cochlear Implants, Hearing health services, Parental reactions

## Abstract

**Background**: The COVID-19 pandemic has affected the world in an unprecedented manner. It has aggravated psychological distress in parents of children with cochlear implants. Continuous use of a speech sound processor is critical for auditory stimulation in children with cochlear implants. However, movement restrictions imposed have affected access to hearing healthcare services. The current study explores the impact of the COVID-19 pandemic on hearing healthcare access for children with cochlear implants.

**Methods**: An online questionnaire survey was conducted among parents of children with cochlear implants.

**Results**: A total of 24 parents responded to the questionnaire. All the respondents reported that COVID-19 has a significant impact on access to hearing health services for their children. Speech processor breakdown and disconnection from the auditory mode of communication had a critical influence on behavioral changes in children.

**Conclusions**: The current study highlights the hurdles faced by the parents in order to access hearing health services for their children. The use of innovative methods such as remote tele-audiology will be the way forward to tackle challenges faced by the parents.

## Introduction

Hearing is one of the most important senses in humans. Auditory development during a young age is critical for the acquisition of normal speech and language development ^1^. Pediatric hearing loss constitutes one of the most important public health challenges ^2^. Children with hearing loss are identified early and habilitated via hearing aids or with cochlear implants (CIs). Continuous auditory stimulation without any interruptions is essential for the successful attainment of language acquisition. Appropriate care and maintenance as well as continuous auditory verbal therapy (AVT) are also essential in attaining these goals ^3^. These are managed by providing seamless access to hearing health services without interruption and restrictions.

However, during the past six months, from the beginning of the year 2020, the world has witnessed an unprecedented attack on humans by a novel virus. The virus was proven to cause acute respiratory disease; the virus was later named severe acute respiratory syndrome coronavirus 2 (SARS-CoV-2) ^4^ and the disease coronavirus disease 2019 (COVID-2019). Towards the end of January 2020, the World Health Organization announced COVID-19 to be a public health emergency of great concern. This was followed by the announcement of stay at home orders and various precautionary measures enforced by various health authorities in order to contain the spread of the virus.

Though the pandemic has had a profound psychological impact on all ^5^, pediatric patients with CIs require additional attention to keep up with their communication needs ^6^. Children with hearing loss pose significant challenges to their parents, particularly when there is limited access to their hearing care providers. The break in the routine of their hearing and therapy follow-up services has had considerable effects on the children as well as their parents ^7^. Hence, the current study aims to explore the impact of the COVID-19 pandemic on hearing healthcare services for children with CIs.

## Methods

### Ethical statement

Ethical approval was obtained for the current study from the Ethics and Research committee of the University Hospital Sharjah (UHS-HERC- 034-20052020) *.* All participants were contacted via telephone and verbal consent was obtained for participation in the study and publication of data. ** Owing to the current pandemic situation, movement restrictions and considering the safety of the participants during the study period, telephonic consent was obtained after thoroughly explaining the consent script. Once the script had been read to the participant, the authors recorded the participant’s agreement or disagreement to consent on the script sheet. This was approved by the Ethics and Research committee of the University Hospital Sharjah (UHS-HERC- 034-20052020) after review of the consent script.

### Study design

A cross-sectional study design was employed for the current study. This study was carried out at the audiology unit of University Hospital Sharjah, United Arab Emirates, for a period of two months from May 2020 to June 2020.

### Study sample

Convenience sampling strategy was used to recruit participants during the study period. The study was targeted towards parents of CI children residing in the region close to the study center. To establish contact with the relevant study participants, professionals such as audiologists, speech language pathologists, auditory verbal therapists (AVTs) and otolaryngologists were identified and contacted in the region. These professionals were asked to share the survey with the desired parents of CI children. They contacted the potential participants through text messages with a link to the survey on.

### Questionnaire development and administration

A questionnaire was developed (see *Extended data*) ^8^ to gather answers to the research questions after discussion and mutual consensus from the authors. The authors’ background includes the specialties: audiology, speech language pathology, AVT and otolaryngology. The questionnaire consists of ten questions and was hosted on SurveyMonkey. The nature of the questions was equally divided into two categories. Five questions were focused on the challenges expressed by the parents. The other five questions were focused on CI user related challenges.

However, the two categories of questions were presented in the questionnaire in a random manner. The questionnaire was prepared in English and translated into an Arabic language version for better comprehension by the respondents. Finally, backward translation was done from Arabic to English to assess any discrepancies in the translation of the questions.

As part of the piloting and face validity testing process, the questionnaire link was shared via text message with five parents of CI children. These participants were randomly selected and the feedback messages obtained from the parents were analyzed by the authors. Minor revisions were made to questionnaire based on the parent’s feedback. Two questions had ambiguous phrases, which were modified and reformulated in the final version of the questionnaire, for better comprehension of the parents.

The questionnaire was self-administered in nature, with a completion duration of less than five minutes. All the questions were created and presented in simple language to the parents of CI children. Care was taken while formulating the questions to capture the parents’ current feelings during the pandemic without referring to their past experiences. This would have been detrimental to exploring their obstacles owing to the current pandemic situation. A Likert response scale was used, in which parents were given the following options: 1) Strongly disagree, 2) Disagree, 3) Neither agree nor disagree, 4) Agree, 5) Strongly agree *.* The survey link was shared with the parents via text message. The participation of the parents was completely on a voluntary basis.

### Statistical analysis

The obtained categorical data was descriptively analyzed and expressed in percentages using Microsoft Excel (2019). For the ease of analysis, the response constructed with the five alternatives were modified into three categories: strongly agree and agree were grouped and coded as 1) agree; category 2) was neither agree nor disagree; and options disagree and strongly disagree were categorized as 3) disagree. Finally, the analyzed data was presented in tables and graphs.

## Results

A total of 31 parents of CI children were initially approached for the study. Out of these, 24 parents responded to the questionnaire sent to them ( Table 1). All the CI users had pre-lingual deafness and the CI had been implanted for longer than one year at the time of the participation in the study.

**Table 1.** Total responses of parents of children with cochlear implants.

**Questions**	**Agree (%)**	**Neither agree** **nor disagree (%)**	**Disagree (%)**	**Total (%)**
**Q1**	**100%**	**0%**	**0%**	**100%**
**Q2**	**96%**	**4%**	**0%**	**100%**
**Q3**	**79%**	**4%**	**17%**	**100%**
**Q4**	**29%**	**50%**	**21%**	**100%**
**Q5**	**96%**	**0%**	**4%**	**100%**
**Q6**	**67%**	**25%**	**8%**	**100%**
**Q7**	**88%**	**8%**	**4%**	**100%**
**Q8**	**96%**	**0%**	**4%**	**100%**
**Q9**	**42%**	**46%**	**12%**	**100%**
**Q10**	**71%**	**21%**	**8%**	**100%**

**Figure 1.**
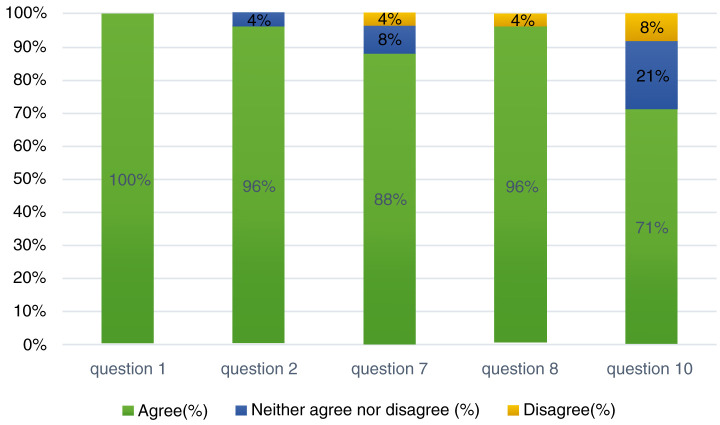
Percentage of responses to the questions related to the challenges faced by the parents.

Of the questions relating to the parents, all of the parents (100%) reported that the COVID-19 pandemic has had an impact on availing timely hearing healthcare services for their children ^8^. 96% of the parents reported that they could not follow up with their CI mapping dates with their centers. It was interesting to note that 88% of the parents felt that the COVID-19 pandemic has been psychologically distressing for them. However, 8% of them neither agreed nor disagreed with that statement, with 4% reporting that they totally disagree that psychological distress was caused by the pandemic. With respect to the home training programs and remote learning aspects, 96% of the parents agreed that the home training methods were challenging and 71% of the parents expressed that remote learning lessons were difficult for their children ( Table 2, Figure 1).

**Table 2.** Challenges expressed by the parents.

**Questions**	**Agree (%)**	**Neither agree** **nor disagree (%)**	**Disagree (%)**	**Total (%)**
**Q1**	**100%**	**0%**	**0%**	**100%**
**Q2**	**96%**	**4%**	**0%**	**100%**
**Q7**	**88%**	**8%**	**4%**	**100%**
**Q8**	**96%**	**0%**	**4%**	**100%**
**Q10**	**71%**	**21%**	**8%**	**100%**

**Figure 2.**
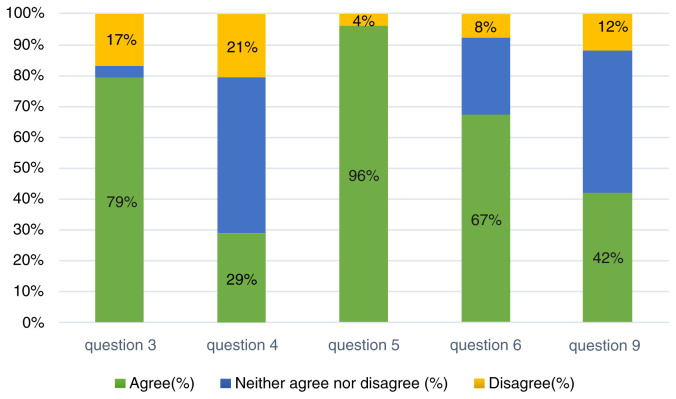
Percentage of responses to the questions related to the cochlear implant user related challenges.

Challenges pertaining to the CI users were also reported and seemed to be drastically affecting the parents. With respect to the speech processor breakdown, 79% of the parents agreed that it affected the auditory communication with the child, whilst 17% disagreed. Interestingly, daily usage of the speech processor during the daytime was reported to be neutral by 50%, and 29% agreed that they used the speech processor adequately. However, regarding the access to auditory training sessions, 96% of the parents reported that they had difficulty in accessing the services. 67% agreed that behavioral changes were common during the stay at home restriction period, with 8% disagreeing with this. The home training programs provided by their clinicians or AVTs were reported to have been followed accurately by 50% of the parents. However, significant number of parents reported that they did not follow the methods at home ( Table 3, Figure 2).

**Table 3.** Responses to the cochlear implant user related challenges.

**Questions**	**Agree (%)**	**Neither agree nor** **disagree (%)**	**Disagree (%)**	**Total (%)**
**Q3**	**79%**	**4%**	**17%**	**100%**
**Q4**	**29%**	**50%**	**21%**	**100%**
**Q5**	**96%**	**0%**	**4%**	**100%**
**Q6**	**67%**	**25%**	**8%**	**100%**
**Q9**	**42%**	**46%**	**12%**	**100%**

## Discussion

The current study aims to understand the impact of COVID-19 on access to hearing healthcare services for children with CI. The results of the study underline the fact that the current pandemic situation has significantly affected children with CI.

It is an undisputable fact that hearing healthcare access is a complex issue which involves an interplay of multiple factors from both parents as well as care providers ^2, 7^. Pediatric hearing loss has already proven to be a challenge for the parents during the pre – pandemic times. Parents of CI children enrolled in the current study reported that they faced issues with not receiving timely hearing health services for their children. These are mainly related to the break in CI mapping follow-up schedules and speech processor break down. It was reported extensively in the literature that for a successful outcome with CI, continuous usage of the speech processor is critical ^9– 11^. In addition, optimal current levels are necessary for the development of auditory sensation ^12, 13^. It is also evident from the current study that the present situation is psychologically pressing for both parents as well as for their children.

Parents have also reported that home training activities were difficult. This could be attributed to the boredom faced by both children and parents due to being confined at home for a long duration. In such scenarios, the child may not be able to cooperate with the task to achieve the target goal assigned by their clinician. Moreover, they faced greater trouble adapting to the remote learning environment.

It also worth noting that CI user related issues caused an additional burden on the parents. For example, in the current study, 79% of the parents reported that speech processor breakdown affected their auditory mode of communication with the child. It is also important to consider that not all CI users will have spare CI processors readily available at home for replacement ^14^. This will add to the psychological anxiety of both parents and their children. It is documented in literature that a breakdown of the CI processor or temporary discontinuation of speech processor usage is strongly correlated with expected outcomes in CI children ^7^. Interestingly, our results suggest that in the current situation, significant behavioral changes in CI children are shown. This can also be attributed to the lack of access to an auditory mode of communication ^15, 16^. Such global changes in behavior and emotional aspects of parents as well as children will have severe impacts on following the home training programs planned by their clinicians.

The findings from our study focused on the enhanced need for the timely access to hearing health services for CI children. The study also shed light on the various needs and hurdles faced by parents. One of the critical aspects of improving service accessibility is reaching out to users through innovative methods. This includes tele-audiology services ^17– 19^, in which the healthcare professionals can provide remote mapping and necessary trouble shooting for the speech processors. Also, a tele-AVT program is another option for parents to engage the child with therapists. This will ensure better compliance and continuity of the care plan. In addition, this will facilitate containment of behavioral changes in children to a certain extent.

During these unprecedented times, hearing health services can also be availed via homecare visits or mobile hearing health services. However, such services are not widely available. In addition, precautionary measures are critical for both children and healthcare professionals before organizing the home visits.

## Conclusions

The COVID-19 pandemic has been reported to have a considerable impact on access to the hearing health services availed by CI children. Parents and CI children are distressed due to the lack of access to services and consequent breakdown in communication. Results from the current study suggest the need for more innovative methods to be employed to meet the needs of CI children. However, the current study has a limitation due to the restricted sample size of participants. Future studies can be carried out by focusing on a wider CI population and their specific needs.

## Data availability

### Underlying data

Figshare: Impact of COVID-19 on the access to hearing health care services for children with cochlear implants: a survey of parents. https://doi.org/10.6084/m9.figshare.12503510.v3 ^8^

This project contains the following underlying data:

- Ayas data.xlsx (Questionnaire data in Microsoft Excel format)

### Extended data

Figshare: Impact of COVID-19 on the access to hearing health care services for children with cochlear implants: a survey of parents. https://doi.org/10.6084/m9.figshare.12503510.v3 ^8^

This project contains the following extended data:

- Extended data-Questionnaire.docx

Data are available under the terms of the Creative Commons Attribution 4.0 International license (CC BY 4.0).
