# Supplementary material for: Impact of COVID-19 on the access to hearing health care services for children with cochlear implants: a survey of parents
Source: F1000Res. 2020 Jul 9;9:690. [Version 1] doi: 10.12688/f1000research.24915.1 (PMC7376500; doi:10.12688/f1000research.24915.1)
Supplement: Peer review history

# Peer review: Reviewer response for version 1

**DOI:** 10.5256/f1000research.27488.r66848

**Published:** 2020-07-22

**Authors:** Holly S. Kaplan, Alison Morrison

**Affiliations:** 1 Speech and Hearing Clinic, Department of Communication Sciences and Special Education, University of Georgia, Athens, GA, USA; 2 UGA Speech and Hearing Clinic, Department of Communication Sciences and Special Education, Mary Frances Early College of Education, Athens, GA, USA

- I found the reference list to be timely and provides citations to give evidence to statements given. Older references would be viewed as more seminal in the field or were written by known experts. I would remove the sentence in the abstract that states parents are experiencing aggravated psychological distress. Your paper found that to be true based on your sample, but I do not believe there are other papers out already that have found the same issues. I would re-word that psychological distress may be a consequence. 
- The study design is appropriate and the questionnaire includes questions that pair to demonstrate consistency in the study. This study could be easily replicated. It would be interesting to do so in different countries or in one country with differing access to CI care. I would also want to re-do this study with the same group post covid. Do the questions on daily use change? 
- I noted 'partly' on the reproducibility as the reader must go to another link to see the actual questions and the data. I would recommend giving each question a key word title, e.g., Q1 could be called Q1Access and Q2Map. This would make reading the tables easier and clearer. 
- I would also look into further statistical analysis of the data.  Are there significant differences in and between question responses? An analysis to determine which questions actually match to each other would also be interesting. Length of implant use, age of child, number of children in family etc. may also impact the answers. I noted you felt the N was low in your group, but I commend you in finding this many parents who are willing to complete the survey during these difficult times. 
- The source data is available, but the reader must of course go to the website where it is located. The conclusions are conservative and reflect the data. I appreciate the difficulty experienced by these families and their honesty in noting AR therapy is hard to keep up with when there are so many things changing rapidly. 
- My one concern would be behavior changes ascribed to implant use -- not sure that your question about behavioral changes is necessarily tied to implant use. As an anecdote, most of my friends are commenting on their children's behavior changes as a result of lockdown and social distancing. 
- I commend the authors in getting this study completed and published so quickly and under pandemic conditions. Excellent work that can be expanded on!

The following comments are from Dr. Morrison.

 Reviewer comments:

- The authors are applauded for executing this study during difficult times when recruitment and engagement with participants is challenging. This article is recommended for publication with minor revision. The following comments are provided:
- The authors indicate that “Care was taken while formulating the questions to capture the parents’ current feelings during the pandemic without referring to their past experiences”. Were additional instructions (not printed on the questionnaire) provided to parents? Several of the questions seem quite general in nature and difficult to directly connect to the experience of parenting a child with CI during the pandemic. For example, the authors note that 88% of the parents report that COVID-19 is psychologically distressing for the parent and in the conclusion state that “parents and CI children are distressed due to the lack of access to services and consequent breakdown in communication.” However, the formulation of this question - at least in English - does not necessarily relate the psychological distress to CI issues or access to hearing healthcare but could be due to their own safety, fear for elderly parents, stress related to work or finances, etc. Additional explanation for how the parents were instructed to answer these questions as specifically related to CI would be helpful. If this information was in the telephone script, the inclusion of the script in the appendices should be considered. Alternatively, if the question was intended to probe psychological distress as a whole, rather than related to hearing healthcare, the authors are encouraged to discuss the role of general parent distress on development of the hearing-impaired child. 
- Additionally, question #4 probed whether speech processor use at home was “adequate” – for which 29% of the parents agreed and 50% of parents neither agreed nor disagreed. Elaboration as to whether speech processor use at home was believed to be different during this pandemic time period compared to typical home use would be helpful. The authors describe that 79% of parents reported that speech processor breakdown affected auditory communication with the child. The authors are encouraged to elaborate whether speech processor breakdown was more frequent or more prolonged during this pandemic time. The authors are encouraged to describe the process for replacing/repairing the speech processor in the UAE (i.e. does this require a trip to a hearing healthcare center? – in the reviewer’s home country, it does not). The authors are encouraged to describe in additional detail the current average delay for hearing healthcare services in the study area (i.e. are most appointments delayed by 1-2 weeks, are all hearing-related appointments cancelled indefinitely, etc.) to provide additional context for the international reader.
- Lastly, question 6 asked parents to reflect on behavioral changes in children with CI in the home. The authors note that “in the current situation, significant behavioral changes in CI children are shown. This can also be attributed to the lack of access to an auditory mode of communication.”  The authors are encouraged to describe whether additional instruction existed to inform the parents to reflect on behavior changes specifically related to communication (vs boredom or disruption of routines, for example) or whether they are hypothesizing the link between the two.

Is the work clearly and accurately presented and does it cite the current literature?

Yes

If applicable, is the statistical analysis and its interpretation appropriate?

Partly

Are all the source data underlying the results available to ensure full reproducibility?

Partly

Is the study design appropriate and is the work technically sound?

Yes

Are the conclusions drawn adequately supported by the results?

Yes

Are sufficient details of methods and analysis provided to allow replication by others?

Partly

Reviewer Expertise:

Audiology.

We confirm that we have read this submission and believe that we have an appropriate level of expertise to confirm that it is of an acceptable scientific standard.

# Peer review: Reviewer response for version 1

**DOI:** 10.5256/f1000research.27488.r66847

**Published:** 2020-07-16

**Authors:** Rohit Ravi

**Affiliations:** 1 Department of Audiology and Speech Language Pathology, Kasturba Medical College, Kasturba Medical College, Karnataka, India

The COVID-19 pandemic has hindered access to hearing healthcare. The present study highlights the impact of the same on children with CI. The survey is the need of the hour to find out the challenges and the ways in which this can be adopted to overcome these challenges.

 I appreciate the authors for undertaking this relevant topic, the study is well written and will definitely be beneficial to the readers & scientific community.

 There are a few minor changes I suggest:

1. Title can be modified to include the location of the study as "Sharjah".
2. Also, add the limitation and clinical implication of the study.

Is the work clearly and accurately presented and does it cite the current literature?

Yes

If applicable, is the statistical analysis and its interpretation appropriate?

Yes

Are all the source data underlying the results available to ensure full reproducibility?

Yes

Is the study design appropriate and is the work technically sound?

Yes

Are the conclusions drawn adequately supported by the results?

Partly

Are sufficient details of methods and analysis provided to allow replication by others?

Yes

Reviewer Expertise:

Pediatric Audiology

I confirm that I have read this submission and believe that I have an appropriate level of expertise to confirm that it is of an acceptable scientific standard.
